# Differential Roles of Human Tau Isoforms in the Modulation of Inflammation and Development of Neuropathology

**DOI:** 10.1002/alz.086518

**Published:** 2025-01-03

**Authors:** Brian Spencer, Jennifer Ngolab, Aaron Schueler, Daniel Sung, Robert A Rissman

**Affiliations:** ^1^ Department of Neurosciences, University of California San Diego, La Jolla, CA USA; ^2^ University of Southern California, San Diego, CA USA; ^3^ Veterans Affairs San Diego Healthcare System, San Diego, CA USA

## Abstract

**Background:**

Alzheimer’s disease (AD) is the most common tauopathy and characterized by the progressive accumulation of Aß and tau. Tau is expressed in two major isoforms containing either 3 or 4 c‐terminal repeats labeled as 3R and 4R tau. While these two isoforms occur in roughly equimolar ratios in AD, most research focus and mouse models of tau center only the 4Rtau protein and not 3Rtau. We recently generated a 3Rtau transgenic mouse to model Pick’s disease which is characterized by the preferential accumulation of 3Rtau. To generate a more human representative model of AD tauopathy and understand specific tau isoform‐mediated neurodegeneration, we generated a transgenic mouse line expressing both 4Rtau and 3Rtau and determined how this impacted the timing and severity of neuropathological and behavioral changes.

**Method:**

3Rtau‐tg and 4Rtau‐tg mice were crossed to generate 3R/4Rtau‐tg bigenic mice. At 3, 6, and 9 months of age, mice were assessed for learning and memory (Morris water maze), cognitive ability and depression (nesting) and hyperactivity (open field). After behavior assessment, mice were sacrificed, and brains were analyzed by IHC for tau accumulation, neuronal loss, and gliosis. Frozen brains were also analyzed for tau protein accumulation by immunoblot and RNA expression by qPCR.

**Result:**

3R/4Rtau bigenic mice expressed increased tau and phosphorylated tau in the hippocampus and cortex compared to 3R or 4R transgenic cohorts as early as 3 months of age. In addition to increased tau pathology, increased astrogliosis and microglia was seen in the bigenic mouse compared to single transgenic tau mice. Finally, the bigenic mouse had significantly greater behavioral deficits compared to either 3Rtau or 4Rtau‐tg mice in spatial learning and memory as well as reduced nest building indicative of depression and/or cognitive deficits.

**Conclusion:**

This new mouse model of tauopathy better recapitulates the pattern, severity and accumulation of tau and associated neuropathology and behavioral changes observed in human tauopathies such as AD and FTD. This new model should supplant existing single transgenic tau models for validation of therapeutic targets and investigations of novel therapies for ADRD.

